# Heteromeric TRPV4/TRPC1 channels mediate calcium-sensing receptor-induced nitric oxide production and vasorelaxation in rabbit mesenteric arteries

**DOI:** 10.1016/j.vph.2017.08.005

**Published:** 2017-09

**Authors:** Harry Z.E. Greenberg, Simonette R.E. Carlton-Carew, Dhanak M. Khan, Alexander K. Zargaran, Kazi S. Jahan, W.-S. Vanessa Ho, Anthony P. Albert

**Affiliations:** Vascular Biology Research Centre, Molecular & Clinical Sciences Research Institute, St. George's, University of London, Cranmer Terrace, London SW17 0RE, UK

**Keywords:** CaSR, calcium-sensing receptors, EC, endothelial cell, IK_Ca_, intermediate conductance calcium-activated potassium channels, NO, nitric oxide, TRPV4, transient receptor potential vanilloid-4, TRPC1, canonical transient receptor potential channel 1

## Abstract

Stimulation of calcium-sensing receptors (CaSR) by increasing the external calcium concentration (Ca^2 +^]_o_) induces endothelium-dependent vasorelaxation through nitric oxide (NO) production and activation of intermediate Ca^2 +^-activated K^+^ currents (IK_Ca_) channels in rabbit mesenteric arteries. The present study investigates the potential role of heteromeric TRPV4-TRPC1 channels in mediating these CaSR-induced vascular responses. Immunocytochemical and proximity ligation assays showed that TRPV4 and TRPC1 proteins were expressed and co-localised at the plasma membrane of freshly isolated endothelial cells (ECs). In wire myography studies, increasing [Ca^2 +^]_o_ between 1 and 6 mM induced concentration-dependent relaxations of methoxamine (MO)-induced pre-contracted tone, which were inhibited by the TRPV4 antagonists RN1734 and HC067047, and the externally-acting TRPC1 blocking antibody T1E3. In addition, CaSR-evoked NO production in ECs measured using the fluorescent NO indicator DAF-FM was reduced by RN1734 and T1E3. In contrast, [Ca^2 +^]_o_-evoked perforated-patch IK_Ca_ currents in ECs were unaffected by RN1734 and T1E3. The TRPV4 agonist GSK1016790A (GSK) induced endothelium-dependent relaxation of MO-evoked pre-contracted tone and increased NO production, which were inhibited by the NO synthase inhibitor L-NAME, RN1734 and T1E3. GSK activated 6pS cation channel activity in cell-attached patches from ECs which was blocked by RN1734 and T1E3. These findings indicate that heteromeric TRPV4-TRPC1 channels mediate CaSR-induced vasorelaxation through NO production but not IK_Ca_ channel activation in rabbit mesenteric arteries. This further implicates CaSR-induced pathways and heteromeric TRPV4-TRPC1 channels in regulating vascular tone.

## Graphical abstract

**Figure:**
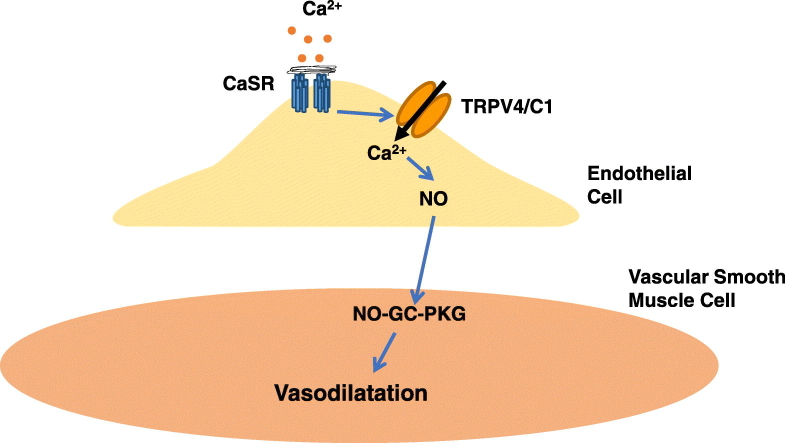

## 1 Introduction

Stimulation of plasmalemmal calcium-sensing receptors (CaSR) by an increase in the extracellular Ca^2 +^ concentration ([Ca^2 +^]_o_) is involved in maintaining plasma Ca^2 +^ homeostasis through the regulation of parathyroid hormone synthesis and secretion from the parathyroid gland, intestinal Ca^2 +^ absorption, and renal Ca^2 +^ excretion [6], [7], [27]. It is also increasingly apparent that CaSR are expressed in tissues not involved in plasma Ca^2 +^ homeostasis, including the cardiovascular system [42], [49], [60].

In the vasculature, functional expression of CaSR in perivascular nerves, endothelial cells (ECs), and vascular smooth muscle cells (VSMCs) is proposed to regulate vascular tone, and may be potential targets for controlling blood pressure [2], [9], [24], [28], [30], [32], [55], [58], [59]. In the presence of closely regulated plasma Ca^2 +^ levels, stimulation of CaSR in the vasculature is considered physiologically possible as localised [Ca^2 +^]_o_ is likely to rise sufficiently at the surface of cells due to active Ca^2 +^ transport mechanisms such as the Ca^2 +^-ATPase and the Na^+^-Ca^2 +^ exchanger, as well as opening and closing of voltage-dependent Ca^2 +^ channels [16], [27], [28], [40], [44]. There is currently little consensus on the function of CaSR in the vasculature, with findings suggesting that stimulation of CaSR induce both vasoconstriction and vasorelaxation through diverse cellular mechanisms [9], [16], [24], [28], [30], [57], [58], [60].

We recently reported that stimulation of CaSR by increasing [Ca^2 +^]_o_ induces an endothelium-dependent vasorelaxation in rabbit mesenteric arteries, which required stimulation of the nitric oxide (NO)-guanylate cyclase (GC)-protein kinase G (PKG) pathway coupled to activation of large conductance Ca^2 +^-activated K^+^ (BK_Ca_) channels in VSMCs, and activation of intermediate conductance Ca^2 +^-activated K^+^ (IK_Ca_) channels inducing endothelium-derived hyperpolarisations [24]. It is unclear how stimulation of CaSR induces these mechanisms, but as endothelium NO synthase (eNOS) and IK_Ca_ channel activation both require a rise in intracellular Ca^2 +^ concentration ([Ca^2 +^]_i_) [10], [11], it seems highly plausible that Ca^2 +^ influx mechanisms are involved. This question forms the focus of the present study.

The transient receptor potential (TRP) superfamily of Ca^2 +^-permeable cation channels form ubiquitously expressed Ca^2 +^ influx pathways, and several TRP channels are functionally expressed in ECs [19], [20], [21], [22], [29], [37], [43], [45], [53], [54], [63]. In particular, there is increasing evidence indicating that TRPV4 channels have a major role in regulating vascular tone, including mediating flow- and agonist-induced vasodilatations via stimulation of NO production and IK_Ca_ channel activation in ECs [3], [4], [8], [18], [26], [37], [38], [51], [52]. It has also been proposed that TRPV4-mediated vascular responses are mediated by heteromeric TRPV4-TRPC1 channel structures expressed in ECs [17], [33], [34], [35], [36], [64]). Therefore, the present work investigates the role of TRPV4, TRPC1, and possible heteromeric TRPV4-TRPC1 channels in CaSR-induced vasorelaxation in rabbit mesenteric arteries. From our findings using wire myography, fluorescent microscopy, and electrophysiological techniques, we propose that heteromeric TRPV4-TRPC1 channels mediate CaSR-induced vasorelaxation and NO production but are not involved in CaSR-induced IK_Ca_ channel activation.

## 2 Methods

### 2.1 Animals

In this study, male New Zealand white rabbits aged 12–16 weeks and weighing 2.5–3 kg were used to examine vascular CaSR mechanisms previously investigated [24]. Rabbits were sourced from Highgate Farm, Louth, United Kingdom. The animals were housed in the Biological Research Facility (BRF) at St George's University of London according to the requirements of the Code of Practice for animal husbandry contained within the Animals Scientific Procedures Act 1986 as amended in 2012. Rabbits were socially housed in pairs and provided with appropriately-sized multi-compartment cages. Room environmental conditions were controlled by an automated building management system that maintained a light:dark cycle of 12:12 h, a room ambient temperature within a range of 18–22 °C, and a relative humidity of 50 ± 10%. Rabbits received ad lib fresh water, a daily allowance of laboratory maintenance rabbit diet, and irradiated hay as an additional source of dietary fibre (Specialist Dietary Services (SDS) UK). Rabbits were killed within 2–4 weeks of arrival by intravenous injection of sodium pentobarbitone (120 mg kg^− 1^) into the peripheral ear vein in accordance with Schedule I of the UK Animals Scientific Procedures Act, 1986 and St George's University of London Animal Welfare and Ethical Review Committee.

### 2.2 Cell and vessel segment preparation

Second-order branches of rabbit superior mesenteric artery were dissected and cleaned of adherent tissue in physiological salt solution (PSS) containing (mM): NaCl 126, KCl 6, Glucose 10, HEPES 11, MgCl_2_ 1.2, and CaCl_2_ 1.5, with pH adjusted to 7.2 with 10 M NaOH. Following dissection, vessels were either cut into 2 mm segments for wire myography studies or were enzymatically dispersed to obtain freshly isolated ECs. To isolate single ECs, vessels were washed in PSS containing 50 μM [Ca^2 +^]_o_ for 5 min at 37 °C and placed in collagenase solution (1 mg ml^− 1^) for 14 min at 37 °C. The vessels were then triturated in fresh PSS and the cell-containing solution was collected and centrifuged for 1 min at 1000 rpm. The supernatant was removed and the cells re-suspended in fresh PSS containing 0.75 mM [Ca^2 +^]_o_, plated onto coverslips, and left at 4 °C for 1 h before use.

### 2.3 Immunocytochemistry

Freshly dispersed ECs were fixed onto borosilicate coverslips with 4% paraformaldehyde (Sigma-Aldrich, Gillingham, UK) for 10 min, washed 3 times with phosphate-buffered saline (PBS), and permeabilised with PBS containing 0.1% Triton X-100 for 20 min at room temperature. Cells were then washed 3 times with PBS and incubated with PBS containing 1% bovine serum albumin (BSA) for 1 h at room temperature. The cells were then incubated overnight at 4 °C with goat-TRPV4 antibodies (1:200, Santa Cruz, Sc47-525) and T1E3, a rabbit anti-TRPC1 antibody generated by GenScript (Piscataway, NJ, USA) using a peptide sequence from a characterised putative extracellular pore region of the TRPC1 subunit [61]. The cells were then washed 3 times with PBS and incubated with secondary antibodies conjugated to fluorescent probes, Alexa Fluor 546-conjugated donkey anti-goat antibody (1:500) or Alexa Fluor 488-conjugated donkey anti-rabbit antibodies (1:500; Thermo Fisher Scientific, Walham, MA, USA). Unbound secondary antibodies were removed by washing with PBS, and nuclei were labelled with 4′,6-diamidino-2-phenylindole (DAPI) mounting medium (Sigma-Aldrich). In control experiments, cells were incubated without primary or secondary antibodies. Cells were imaged using a Zeiss LSM 510 laser scanning confocal microscope (Carl Zeiss, Jena, Germany). Excitation was produced by 546 nm or 488 nm lasers and delivered to the specimen via a Zeiss Apochromat × 63 oil-immersion objective. Emitted fluorescence was captured using LSM 510 software (release 3.2; Carl Zeiss). Two-dimensional images cut horizontally through the middle of the cells were captured and raw confocal imaging data processed using Zeiss LSM 510 software. Final images were produced using PowerPoint (Microsoft XP; Microsoft, Richmond, WA, USA).

### 2.4 Proximity ligation assay

Freshly isolated ECs were studied using the Duolink in situ proximity ligation assay (PLA) detection kit 563 (Olink, Uppsala, Sweden) [50]. Cells were plated onto coverslips, fixed with PBS containing 4% paraformaldehyde for 15 min, and permeabilized in PBS containing 0.1% Triton X-100 for 15 min. Cells were blocked for 1 h at 37 °C in blocking solution, and incubated overnight at 4 °C with anti-TRPV4 and T1E3 antibodies (both at dilution 1:200) in antibody diluent solution. Cells were labelled with combinations of either anti-goat PLUS/anti-rabbit MINUS 1 h at 37 °C. Hybridized oligonucleotides were ligated for 30 min at 37 °C prior to amplification for 100 min at 37 °C. Red fluorescence-labelled oligonucleotides were then hybridized to rolling circle amplification products, and visualized using a Confocal LSM 510 (Carl Zeiss).

### 2.5 Isometric tension recordings

Effects of stimulating CaSR and TRPV4-containing channels on vascular tone were investigated using wire myography. Vessel segments of 2 mm in length were mounted in a wire myograph (Model 610 M; Danish Myo Technology, Aarhus, Denmark) and equilibrated for 30 min at 37 °C in 5 ml of gassed (95% O_2_/5% CO_2_) Krebs–Henseleit solution of the following composition (mM): NaCl 118, KCl 4.7, MgSO_4_ 1.2, KH_2_PO_4_ 1.2, NaHCO_3_ 25, CaCl_2_ 2, D-glucose 10, pH 7.2. Vessels were then normalised to 90% of the internal circumference predicted to occur under a transmural pressure of 100 mmHg [39]. After normalisation, vessels were left for 10 min and were then challenged with 60 mM KCl for 5 min. Endothelium integrity was assessed by stably pre-contracting vessels with 10 μM methoxamine followed by the addition of 10 μM carbachol (CCh). Vessels in which CCh-induced relaxations were > 90% of pre-contracted tone were designated as having a functional endothelium. When required, endothelium was removed by rubbing the intima layer with a human hair and CCh-induced relaxations of < 10% of pre-contracted tone indicated successful removal. Vessel segments were incubated for 30 min in fresh Krebs solution containing 1 mM CaCl_2_ and then pre-contracted with 10 μM methoxamine as required. This was followed by cumulative additions of CaCl_2_, increasing [Ca^2 +^]_o_ between 1 and 6 mM, or 10 nM GSK1016790A in the presence of inhibitors tested or their respective vehicles. All inhibitors were added to the vessel segments 30 min before the construction of concentration-response curves to [Ca^2 +^]_o_ or GSK1016790A. For each experiment, vehicle controls were performed using vessel segments from the same animal.

### 2.6 NO imaging

ECs were placed in a sterilised 96-well plate and left for 1 h at 4 °C. Cells were loaded with the NO fluorescent dye DAF-FM diacetate (1 μM), incubated at 4 °C for 20 min and then washed with PSS containing 1 mM [Ca^2 +^]_o_. The cells were then left for another 30 min at 4 °C to allow complete de-esterification of intracellular diacetate. Inhibitors tested or their respective vehicles were also added at this point. Changes in fluorescence following 5 min of CaSR stimulation with 6 mM [Ca^2 +^]_o_, 10 nM GSK1016790A, or 10 μM capsaicin were captured using a Zeiss Axiovert 200 M Inverted microscope and processed and analysed using AxioVision SE64 Software (Rel. 4.9.1; Carl Zeiss).

### 2.7 Electrophysiology

Whole-cell and perforated-patch clamp configurations were used to record K^+^ conductances and single cation channel currents were measured using cell-attached patches. Recordings were made with an Axopatch 200B amplifier (Axon Instruments, Union City, CA, USA) at room temperature (20–23 °C). Whole-cell and perforated-patch currents were filtered at 1 kHz (− 3 dB, low-pass 8-pole Bessel filter, Frequency Devices model LP02; Scensys, Aylesbury, UK) and sampled at 5 kHz (Digidata 1322A and pCLAMP 9.0 software; Molecular Devices, Sunnydale, CA, USA), whereas single cation channel currents were filtered at 100 Hz and sampled at 1 kHz.

Whole-cell K^+^ currents were evoked by dialysing cells with a pipette solution containing 3 μM free Ca^2 +^ and perforated-patch K^+^ currents were induced by bath applying 6 mM [Ca^2 +^]_o_. Current/voltage relationships (I/V) were obtained by applying a 200 ms voltage ramp from − 100 mV to + 100 mV every 30 s from a holding potential of − 60 mV. The external bathing solution for both whole-cell and perforated-patch recordings contained (mM): NaCl 134, KCl 6, Glucose 10, HEPES 10, MgCl_2_ 1, CaCl_2_ 1 (adjusted to pH 7.4 with 10 M NaOH). For whole-cell recordings, the pipette solution contained (mM): KCl 134, HEDTA 5, HEPES 10, MgCl_2_ 5.53 (1 mM free Mg^2 +^) and CaCl_2_ 0.207 (3 μM free Ca^2 +^) (pH 7.2). The amounts of MgCl_2_ and CaCl_2_ added were determined using EqCal software (Biosoft, Cambridge, UK). For perforated-patch recordings the pipette solution contained (mM): K-aspartate 110, KCl 30, NaCl 10, HEPES 10, MgCl_2_ 1, pH 7.2 with 10 M NaOH, and amphotericin (200 μg ml^− 1^). The external bathing solution for cell-attached patch recordings contained (mM): 126 KCl, 1 CaCl_2_, 10 HEPES, and 11 glucose, adjusted to pH 7.2 with 10 M KOH. The patch pipette solution contained (mM): 126 NaCl, 1 CaCl_2_, 10 HEPES, and 11 glucose adjusted to pH 7.2 with 10 M NaOH. 100 μM DIDS, 100 μM niflumic acid, 10 mM TEA, 100 nM Apamin (Apa), and 100 nM Charybdotoxin (CbTX) were also included in the patch pipette solution to block Ca^2 +^ and swell-activated Cl^−^ conductances, voltage-gated K^+^ channels, and SK_Ca_, IK_Ca_, and BK_Ca_ channels respectively. This enabled cation conductances to be recorded in isolation. Single cation channel currents were activated by including 10 nM GSK1016790A in the patch pipette solution.

### 2.8 Data and statistical analysis

All data presented are mean ± SEM and for all experiments, P < 0.05 was considered a significant difference between groups. For whole cell and perforated patch clamp recordings, data were analysed using 2-way ANOVA, comparing the effect of increasing voltage on membrane current in treated vs. control cells. Figures and analyses were made using MicroCal Origin 6.0 software (MicroCal Software, Northampton, MA, USA). For wire myography experiments, all relaxant responses are expressed as percentage relaxation of tension induced by 10 μM methoxamine. Responses to increasing [Ca^2 +^]_o_ in treated vs. control vessels were analysed by 2-way ANOVA followed by Bonferroni post hoc tests. Bonferroni comparisons are shown above the graph data points whereby: *P < 0.05, **P < 0.01, ***P < 0.001, ****P < 0.0001 vs. control. For GSK1016790A-induced responses, data were compared using One-way ANOVA. Statistical analyses, including calculation of EC_50_ and E_max_ values, and all graphs were made using Graphpad Prism 6 software (GraphPad Software, Inc., San Diego, CA, USA). For NO imaging experiments, changes in fluorescence were quantified by selecting a cell as a region of interest (ROI) and comparing fluorescence levels within the ROI before and after the experimental protocols and analysed using One-way ANOVA. Figures and analysis were made using Graphpad Prism 6 (GraphPad Software, Inc., San Diego, CA, USA).

### 2.9 Materials

All drugs were purchased from Sigma-Aldrich (Sigma Chemical Co., Poole, UK) or Tocris (Tocris Biosciences, Bristol, UK). Drugs were dissolved in distilled water or dimethyl sulfoxide (DMSO).

## 3 Results

### 3.1 TRPV4 and TRPC1 channel proteins are colocalised in rabbit mesenteric artery ECs

**Fig. 1:**
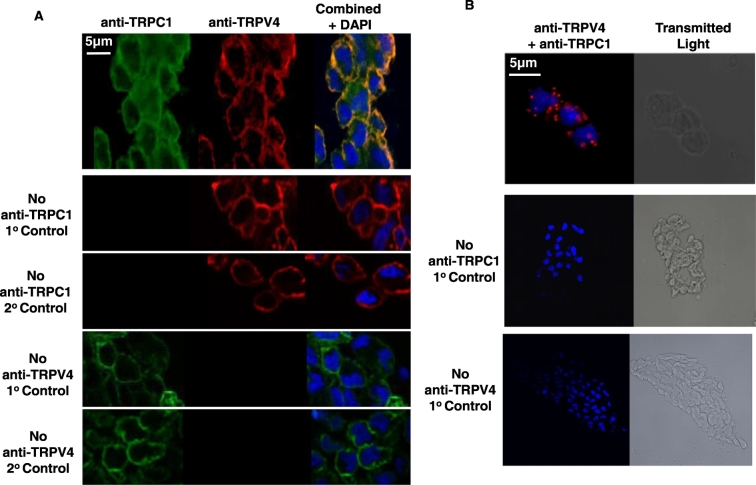
Expression and co-localisation of heteromeric TRPV4-TRPC1 channels in freshly isolated mesenteric artery ECs. A, Representative immunocytochemical images of TRPV4 (red) and TRPC1 (green) proteins in rabbit mesenteric artery ECs, showing expression and co-localisation (yellow) at the plasma membrane. Representative images showing that the absence of primary anti-TRPV4 and anti-TRPC1 antibodies, or their corresponding secondary antibodies failed to produce any immunocytochemical staining. B, Representative images from proximity ligation assays illustrating TRPV4 and TRPC1 co-localisation staining (red) in rabbit ECs. In the absence of primary anti-TRPV4 and anti-TRPC1 antibodies failed to produce any PLA staining.

In our initial experiments, we examined the expression of TRPV4, TRPC1, and potential co-localisation between these two channel proteins in freshly isolated rabbit mesenteric artery ECs. Fig. 1A shows that TRPV4 and TRPC1 proteins were expressed in ECs using immunocytochemistry, with staining and co-localisation present at the plasma membrane. Fig. 1B provides further evidence using proximity ligation assay that TRPV4 and TRPC1 co-localisation signals were present in ECs.

### 3.2 CaSR-induced vasorelaxation and NO production are reduced by TRPV4 and TRPC1 channel inhibitors in rabbit mesenteric artery

In this series of experiments, we investigated the effect of the TRPV4 channel blockers RN1734 and HC067047 [3], [51], [56], and the externally-acting TRPC1 antibody T1E3, which is known to act as a TRPC1 channel blocking agent [46], [61] on CaSR-induced vasorelaxation and NO production.

**Fig. 2:**
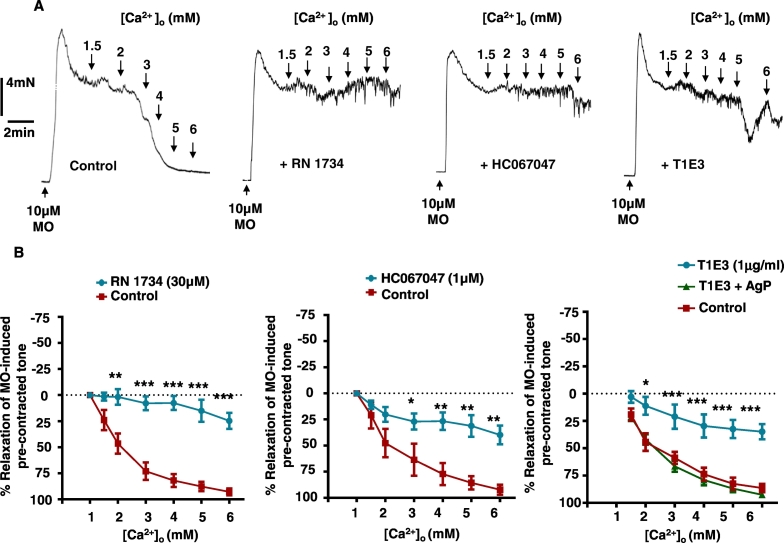
Effect of TRPV4 and TRPC1 blockers on [Ca^2 +^]_o_-induced relaxation in rabbit mesenteric arteries. A Representative traces and B, mean data showing the inhibitory effect of the TRPV4 antagonists RN1734 and HC067047, and the TRPC1 blocker T1E3 on [Ca^2 +^]_o_-induced relaxations of pre-contracted tone. Pre-incubation of T1E3 with AgP prevented the inhibitory action of TIE3. n = 5 animals, 3 vessel segments per animal.

Fig. 2 shows that increasing [Ca^2 +^]_o_ between 1 and 6 mM produced concentration-dependent relaxation of pre-contracted tone induced by 10 μM methoxamine, previously shown to be mediated by stimulation of CaSR [24]. [Ca^2 +^]_o_ -evoked relaxation was reduced following pre-treatment of vessel segments with 30 μM RN1734, 1 μM HC067047, and 1 μg ml^− 1^ T1E3 (Table 1). To show selectivity of the inhibitory response to T1E3, pre-incubation of T1E3 with its antigenic peptide (AgP) prevented application of this antibody attenuating [Ca^2 +^]_o_-induced relaxation (Table 1).

**Table 1:** Effect of various inhibitors tested on [Ca^2 +^]_o_-induced vasorelaxation.

Rabbit	EC_50_ (mM)	E_max_ (%)	N
Control	2.2 ± 0.06	92.7 ± 4.2	8
+ RN1734 (30 μM)	4.3 ± 0.1*	24 ± 7.5*	5
+ HC067047 (1 μM)	2.1 ± 0.04	39.7 ± 8.9*	5
+ T1E3 (1 μg ml^− 1^)	2.8 ± 0.12*	34.7 ± 6.7*	5
+ T1E3 + AgP	2.2 ± 0.07	92.6 ± 1.9	5

Data shown are mean values ± SEM. Data are compared by unpaired Student's *t*-test with *P < 0.05 vs. respective control group considered significant. N = number of animals used, with at least 3 vessel segments used per animal.

**Fig. 3:**
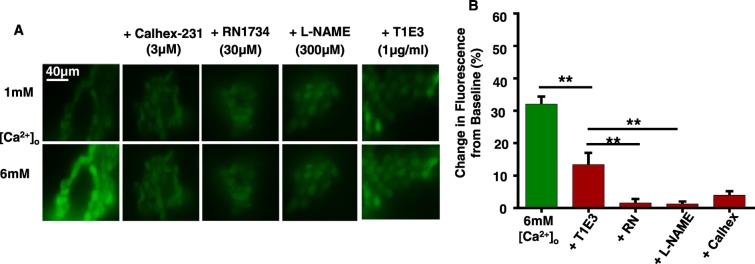
Effect of TRPV4 and TRPC1 blockers on [Ca^2 +^]_o_-induced NO production in rabbit mesenteric arteries. A, Representative images showing that Calhex-231, L-NAME, RN1734, and T1E3 reduced DAF-FM fluorescence induced by 6 mM [Ca^2 +^]_o_ in freshly isolated ECs. B, Mean data showing the effect of 6 mM [Ca^2 +^]_o_ and pre-treatment with Calhex-231, L-NAME, RN1734, and T1E3 on DAF-FM fluorescence. Each experiment from n = 5 animals, > 50 cells per animal.

Fig. 3 reveals that increasing [Ca^2 +^]_o_ from 1 mM to 6 mM potentiated baseline DAF-FM fluorescence by over 30%, which was inhibited by pre-treatment with the calcilytic 3 μM Calhex-231, the NO synthase inhibitor 300 μM L-NAME, and RN1734 and T1E3. It was apparent that RN1734 had a greater inhibitory effect on [Ca^2 +^]_o_-induced vasorelaxation and increases in DAF-FM fluorescence than T1E3.

**Fig. 4:**
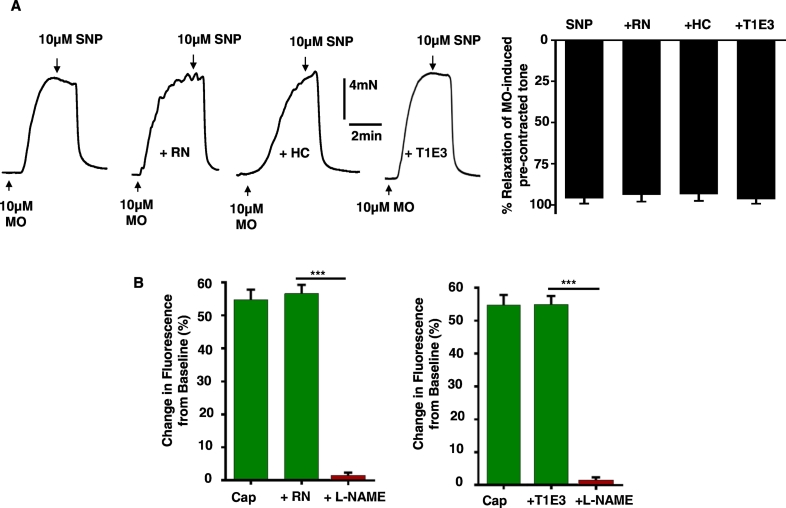
Effect of RN1734, HC067047, and T1E3 on SNP-induced relaxations, and effect of RN1734 and T1E3 on capsaicin-induced NO production. A, Traces and mean data showing that pre-treatment with RN1734, HC067047, and T1E3 had no effect on SNP-induced relaxations of pre-contracted tone in segments of rabbit mesenteric arteries. B, Mean data showing that capsaicin-induced increase in DAF-FM fluorescence were reduced by L-NAME but were unaffected by RN17 and T1E3. n = 5 animals, > 40 cells per animal.

In control experiments, Fig. 4A shows that pre-treatment with RN1734, HC067047, and T1E3 had no effect on relaxations of pre-contracted tone induced by the NO donor 10 μM SNP. In addition, Fig. 4B demonstrates that increases in DAF-FM fluorescence evoked by the selective TRPV1 agonist 10 μM capsaicin were unaffected by RN1734 and T1E3. These results indicate that RN1734, HC067047, and T1E3 do not alter the ability of vessel segments to relax, and that RN1734 and T1E3 do not produce non-specific reductions in NO production.

### 3.3 CaSR-induced IK_Ca_ currents are unaffected by TRPV4 and TRPC1 channel inhibitors in rabbit mesenteric artery ECs

**Fig. 5:**
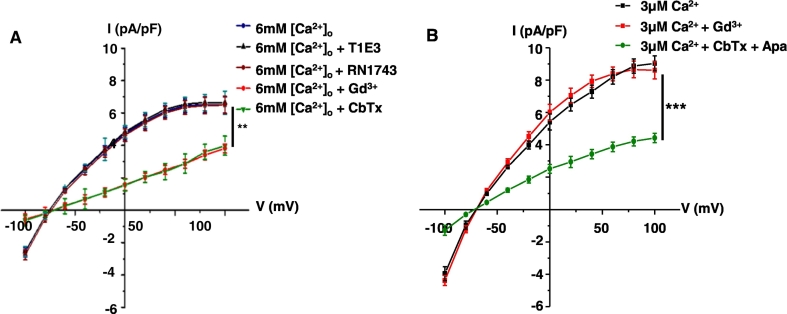
Effect of RN17 and T1E3 on [Ca^2 +^]_o_-induced K^+^ channel currents in freshly isolated rabbit mesenteric artery ECs. A, Mean current/voltage relationships of perforated-patch K^+^ channel currents induced by 6 mM [Ca^2 +^]_o_ showing that currents were inhibited by CbTX and Gd^3 +^ but were unaffected by RN1734 and T1E3. B, Mean current/voltage relationships of whole-cell K^+^ currents induced by inclusion of 3 μM free Ca^2 +^ in the patch pipette solution were inhibited by a combination of CbTX and apamin (Apa) but were unaffected by Gd^3 +^. Each point from 6 patches from n = 5 animals.

Fig. 5A shows that increasing [Ca^2 +^]_o_ from 1 mM to 6 mM evoked a mean macroscopic K^+^ current in freshly isolated ECs using the perforated-patch configuration, which had inward rectification at positive membrane potentials, reversed near to equilibrium potential for K^+^ ions (E_K_ is − 80 mV), and was abolished by the IK_Ca_ channel blocker, 100 nM charybdotoxin (CbTX). These properties are consistent with previous studies demonstrating that stimulation of CaSR activates IK_Ca_ currents [24], [58]. Interestingly, [Ca^2 +^]_o_-induced IK_Ca_ currents were not inhibited by RN1734 and T1E3, but were prevented by the cation channel blocker, and pan-selective TRP channel inhibitor, 100 μM Gd^3 +^[5]. Fig. 5B shows that inclusion of 3 μM free Ca^2 +^ in the patch pipette solution evoked a mean whole-cell K^+^ current which was inhibited by co-application of both CbTX and the small-conductance Ca^2 +^-activated K^+^ channel (SK_Ca_) blocker 100 nM Apamin and therefore composed of IK_Ca_ and SK_Ca_ channels [24], but was unaffected by Gd^3 +^. This indicates that Gd^3 +^ is not directly blocking IK_Ca_ or SK_Ca_ channels but is likely to be blocking a Ca^2 +^ influx pathway.

These results provide pharmacological evidence that channels composed of TRPV4 and TRPC1 are involved in CaSR-induced vasorelaxation and NO production but are unlikely to be required for CaSR-induced IK_Ca_ channel activation.

### 3.4 Vasorelaxations and NO production stimulated by the TRPV4 agonist GSK are reduced by both TRPV4 and TRPC1 inhibitors

**Fig. 6:**
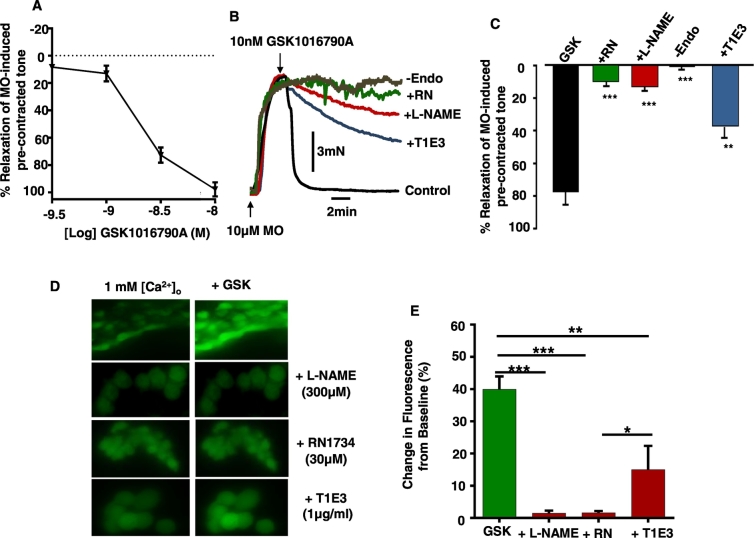
Effect of T1E3 on GSK-induced relaxations of pre-contracted tone and NO production in rabbit mesenteric arteries. A, Mean data showing GSK produced a concentration-dependent vasorelaxation of pre-contracted tone. B & C, Original traces and mean data showing that GSK-induced relaxation of pre-contracted tone was inhibited by removal of endothelium, and L-NAME, RN1734 and T1E3. Each point from n = 5 animals with n = 3 vessel segments from each animal. D & E, Representative images and mean data showing that GSK activated an increase in DAF-FM fluorescence which was reduced by L-NAME, RN17, and T1E3. Each experiment was from n = 5 animals, > 50 cells per animal.

As the present study suggests that heteromeric TRPV4-TRPC1 channels may mediate CaSR-induced vasorelaxation and NO production, we hypothesised that the selective TRPV4 agonist GSK101970A (herein termed GSK) would induce vasorelaxation and NO production which are inhibited by TRPC1 blockade. Fig. 6A, B & C illustrate that GSK produced a concentration-dependent relaxation of pre-contracted tone of rabbit mesenteric artery segments, which were reduced by removal of the endothelium, and by pre-treatment with L-NAME, RN1734, and T1E3. Moreover, Fig. 6D & E also show that GSK induced an increase in baseline DAF-FM fluorescence by about 40% which was attenuated by L-NAME, RN1734, and T1E3. Together, these results indicate that TRPC1 contributes to GSK-induced vasorelaxation and NO production.

### 3.5 GSK activates cation channel activity in ECs which is reduced by both TRPV4 and TRPC1 inhibitors

**Fig. 7:**
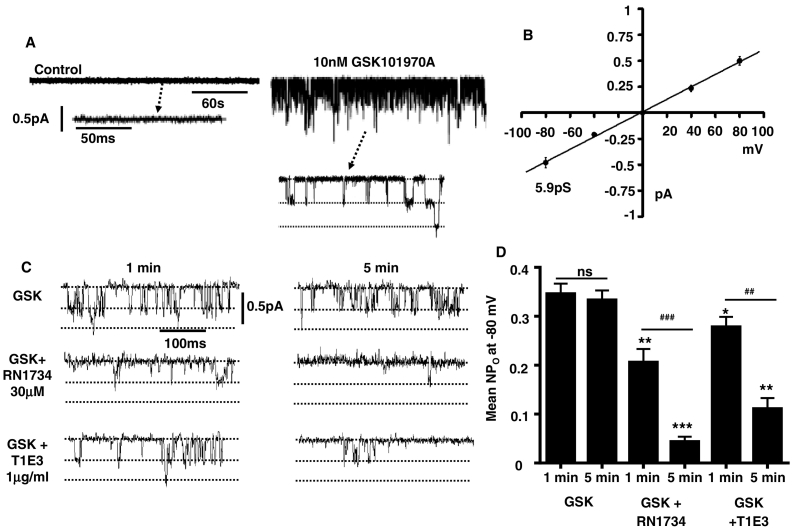
GSK-evoked cation channel currents in rabbit mesenteric artery ECs. A, Application of GSK in the patch pipette solution activates single cation channel activity in cell-attached patches held at − 80 mV. B, Mean current/voltage relationship of GSK-evoked cation channel activities showing channels had unitary conductances of 5.9pS. C, Inclusion of RN1734 and T1E3 in the patch pipette solution inhibited GSK-evoked cation channel activity. D, Mean data showing that RN1734 and T1E3 inhibit mean NP_o_ of GSK-evoked cation channel activity. Each data set from at least 6 patches, from at least n = 5 animals. *P < 0.05, **P < 0.005, ***P < 0.001 vs. respective GSK-only control. ^#^P < 0.05 ^##^P < 0.005 GSK-evoked activity after 1 min vs. after 5 min. in the presence of the inhibitors tested.

In our final experiments, we investigated single TRPV4-containing channel activity in ECs activated by GSK. Fig. 7A & B show that inclusion of 10 nM GSK in the patch pipette solution evoked single cation channel activity in cell-attached patches from ECs, which had similar current amplitudes of about − 0.5pA at − 80 mV that corresponded to unitary conductances of about 6pS. Fig. 7A shows that cation channel activity was not recorded when GSK was absent from the patch pipette solution. Fig. 7C & D reveal that when included on its own, GSK-evoked 6pS cation channel activity was maintained throughout the recording (> 5 min) whereas when either RN1734 or T1E3 were co-applied in the patch pipette solution GSK-evoked cation channel activity was greatly reduced by over 80% and 70% respectively after 5 min.

These results suggest that native TRPV4-containing channels activated by GSK in rabbit mesenteric artery ECs are likely to be composed of a single channel structure with a unitary conductance of 6pS, which is composed of TRPV4 and TRPC1 channel proteins.

## 4 Discussion

The present study proposes that heteromeric TRPV4-TRPC1 channels mediate CaSR-induced vasorelaxation through NO production but not activation of IK_Ca_ channels in rabbit mesenteric artery ECs. Interestingly, our findings suggest that TRPV4-TRPC1 channels with a unitary conductance of 6pS may be the predominant native TRPV4-containing channels in these ECs.

### 4.1 Heteromeric TRPV4-TRPC1 channels mediated CaSR-induced vasorelaxation via NO production

The present study together with our recent findings indicate that stimulation of CaSR by increasing [Ca^2 +^]_o_ induces an endothelium-dependent relaxation of rabbit mesenteric arteries, with a significant contribution involving NO production [24], [25].

Our current results reveal that TRPV4 and TRPC1 proteins are co-localised in ECs, and that CaSR-induced vasorelaxation and NO generation are both inhibited by the TRPV4 inhibitors RN1734 and HC067047, and the TRPC1 blocking antibody T1E3. Our conclusion is further supported by GSK-induced vasorelaxation and NO production also being inhibited by RN1734 and T1E3. These results are in agreement with earlier studies which proposed that a heteromeric TRPV4-TRPC1 channel is expressed in ECs, which is also thought to be composed of TRPP2 subunits [17], [33], [34], [35], [36], [64]. This makes this channel rather unique in that it is composed of subunits from three different subfamilies of the TRP channel superfamily.

In combination with our earlier findings, we propose that stimulation of CaSR activates TRPV4-TRPC1-mediated Ca^2 +^ influx, which leads to Ca^2 +^-CaM-eNOS inducing the classical NO-GC-PKG pathway and vasorelaxation. Thus taken together, these data make an important contribution to our current understanding of how CaSR might regulate vascular tone. Physiologically, it is thought that plasmalemmal Ca^2 +^ pumps and exchangers contribute to significant increases in [Ca^2 +^]_o_ within the local vascular microenvironment, producing extracellular Ca^2 +^ clouds within the vascular interstitium. These Ca^2 +^ clouds then stimulate vascular CaSR to regulate the contractile state of VSMCs as well as the character of endothelial-dependent regulation of vascular tone ([14], [16], [44], [60], [65]). Though the current study uses a range of [Ca^2 +^]_o_ to stimulate CaSR (1–6 mM), future work will be required to establish the precise physiological changes in [Ca^2 +^]_o_ occurring within the vascular microenvironment in order to fully understand how CaSR might regulate vascular tone.

Previous studies have shown that heteromeric TRPV4-TRPC1 channels behave as store-operated channels in ECs, and that TRPC1 confers the ability of TRP channels to be activated by store depletion via STIM1-mediated mechanisms in different cell types [1], [34], [35], [41], [46], [47], [48], [54], [62]. Given that CaSR predominantly couple to Gαq-PLC-IP_3_ signaling when stimulated by [Ca^2 +^]_o_[13], we propose that CaSR-induced heteromeric TRPV4-TRPC1 channel activation might occur downstream of Ca^2 +^ store depletion and the translocation of STIM1 to the channel, though it will be important to clarify the precise mechanism in future work.

TRPC6 channels have been previously linked to CaSR-induced contraction, proliferation and migration of VSMCs in pulmonary arterial hypertension [55], and to CaSR-mediated rises in [Ca^2 +^]_i_ in human aortic VSMCs [12]. However, the present findings provide the first evidence that TRP channels mediate CaSR-induced responses in ECs, representing an important advance in our understanding of how stimulation of CaSR regulates vascular tone. Our data also contributes to the significant evidence that TRPV4-containing channels have critical roles of controlling vascular tone [3], [4], [8], [18], [26], [37], [38], [51], [52].

### 4.2 Heteromeric TRPV4-TRPC1 channels are not required for CaSR-induced IK_Ca_ channel activation

Our previous work showed that in addition to NO generation, CaSR-induced vasorelaxation is also mediated by activation of IK_Ca_ channels in rabbit mesenteric artery ECs which presumably induces endothelium-derived hyperpolarisations [24], [25]. The present work shows that [Ca^2 +^]_o_-induced IK_Ca_ channel activation was not affected by RN1734 and T1E3 indicating that heteromeric TRPV4-TRPC1 channels are unlikely to be involved. However, [Ca^2 +^]_o_-induced IK_Ca_ channel activation was abolished by the cation channel blocker, and pan-TRP channel inhibitor, Gd^3 +^[5]. This poses the intriguing possibility than another TRP channel is coupled to CaSR stimulation, which mediates Ca^2 +^ influx coupled to IK_Ca_ channel activation. A possible candidate is TRPC3, which is expressed in ECs and has been linked to EDH in several different vascular beds [23], [31], [45]. It is possible that CaSR-activated TRP channels may be coupled to distinct functions via different activation pathways. For example, receptor-operated TRP channels such as TRPC3 may be coupled to IK_Ca_ channel activation and relaxation, whereas store-operated TRPV4-TRPC1 channels may be coupled to NO production and relaxation. What is clear is that there is need for future detailed experiments on characterization of CaSR-evoked TRP channels in ECs, their activation pathways, and their vascular function.

### 4.3 Are heteromeric TRPV4-TRPC1 channels the predominant native TRPV4-containing channels in rabbit mesenteric artery ECs?

Our results show that the TRPV4 agonist GSK activated cation channel activity with a unitary conductance of about 6pS in rabbit mesenteric artery ECs, which was inhibited by RN1734 and T1E3. These findings suggest that the predominant native TRPV4-containing channels in these ECs are also composed of TRPC1 subunits forming a heteromeric TRPV4-TRPC1 channel.

In contrast to the present work, over-expression of TRPV4 and TRPC1 subunits and TRPV4-TRPC1 concatamers in HEK293 cells both produced 4αPDD-evoked inward single channel activity which had a unitary conductance of about 80pS [34], [35], which is obviously very different from the 6pS conductance of the channels we recorded. It may be that electrophysiological properties of these channels are different in over-expression systems compared to native cells in their physiological environment. In addition, perhaps the low 6pS conductance also reflects the presence of TRPP2, or other components, which form the native channel. It will be important to investigate these differences in the future.

Throughout this study the TRPC1 blocker T1E3 was not as effective in reducing CaSR-induced vasorelaxations, NO production, and GSK-evoked cation channel activity compared to RN1734. The reason for this is unclear, but it may be because the T1E3 blocking antibody is less potent than a small molecular weight inhibitor. It is unlikely that differences between the effects of T1E3 and RN1734 are due to different populations of TRPV4-containing channels in our ECs, as we clearly show that GSK only activated channels with a single 6pS conductance.

Sonkusare et al. [51] proposed that GSK-activated large amplitude Ca^2 +^ sparklets mediated by Ca^2 +^ influx through opening of a small number TRPV4 channels (cooperative cluster of about 4 channels) produce maximum endothelium-dependent vasorelaxation via stimulation of SK_Ca_ and IK_Ca_ channels, but not NO production, in pressurised 3rd order mouse mesenteric arteries. In contrast, the present work shows that GSK-induced vasorelaxation is mediated by NO generation in 2nd order rabbit mesenteric arteries using wire myography. These disparities may represent differences between species, pressurised vessels and wire myography, and composition and cellular function of TRPV4-containing channels in different order vessels. It would be interesting to investigate if the GSK-activated 6pS TRPV4-TRPC1 channels observed in rabbit mesenteric artery ECs could support sufficient Ca^2 +^ entry to mediate Ca^2 +^ sparklets, and if TRPC1 is involved in GSK-mediated Ca^2 +^ sparklets and vasorelaxations in mouse mesenteric artery ECs. What is clear is that there is considerable evidence that TRPV4 has a significant role in endothelium-dependent regulation of vascular tone in physiological and pathological settings [3], [15], [37], [43], [51], [52], and that further work is needed to elucidate the role of TRPV4-containing channels, including heteromeric TRPV4-TRPC1 structures, as potential therapeutic targets for vascular disease.

## 5 Conclusion

The major finding of this study is that activation of a native heteromeric 6pS TRPV4-TRPC1 channel is involved in CaSR-induced vasorelaxations through NO production in rabbit mesenteric artery ECs. In addition, a distinct TRP-like cation channel is likely to be involved in coupling CaSR stimulation to IK_Ca_ channel activation and vasorelaxation. These results further highlight the importance of CaSR and TRPV4-TRPC1 channels in regulation of vascular tone, which may have potential clinical implications, indicating that CaSR may represent novel therapeutic targets for controlling vascular contractility.

## Funding

This work was supported by a British Heart Foundation PhD Studentship for H. Z. E. Greenberg (FS/13/10/30021 to A.P.A); and by the Biotechnology and Biological Sciences Research Council (BB/J007226/1 to A.P.A).

## Conflict of interest

None declared.

## Author contributions

H.Z.E.G, S.R.E.C-C, D.M.K, K.S.J, and A.K.Z performed and analysed experiments. H.Z.E.G, W-S. V. Ho, and A.P.A conceived the experimental design. H.Z.E.G and A.P.A wrote the manuscript. All authors contributed to the preparation of the manuscript, and critically advised and agreed to the final submitted article.
